# The tip-of-the-tongue state and curiosity

**DOI:** 10.1186/s41235-017-0065-4

**Published:** 2017-07-18

**Authors:** Janet Metcalfe, Bennett L. Schwartz, Paul A. Bloom

**Affiliations:** 1 Columbia University, New York, NY USA; 2 Florida International University, Miami, FL USA

**Keywords:** Tip-of-the-tongue, Curiosity, Google, Metacognition, Region of proximal learning

## Abstract

Theories of study time allocation and of curiosity suggest that people are most engaged with and want to devote their time to materials that are not completely mastered but also are not so difficult that they might be impossible. Their curiosity is thought to be triggered by items that are almost known, or are in what is sometimes called the region of proximal learning. Answers that are on the tip-of-the-tongue (TOT)—not immediately recallable but nevertheless evoking a feeling of imminent recall—seem, intuitively, to be materials that have this characteristic of being almost, but not quite, fully known. We therefore, hypothesized that people would be particularly curious to see the answers to questions for which the answers were on the tips of their tongues. To test the TOT curiosity hypothesis, we gave participants 82 general information questions and quickly asked whether the answers were or were not on the tips of their tongues and whether they wanted to see the answers later. Overwhelmingly, items that were accompanied by a TOT feeling were those which evoked participants’ curiosity, regardless of whether the feeling occurred in conjunction with an error of commission, an error of omission, or even with the correct answer.

**Electronic supplementary material:**

The online version of this article (doi:10.1186/s41235-017-0065-4) contains supplementary material, which is available to authorized users.

## Significance

This article investigates metacognitive conditions that induce people’s need to know. An mTurk survey revealed that people think they are most curious—operationalized, in the survey, as having the strongest urge to Google the answer—when they do not know the answer but not when they know the answer. However, they think they are indiscriminate among ‘not known’ states: when they are sure they do not know, when they have a feeling that they might be able to recognize the answer, and when they are in a genuine ‘tip-of-the-tongue’ (TOT) state. To ascertain whether they were correct that all ‘don’t know’ states were equal, or whether there is a special metacognitive state associated with curiosity, we gave participants 82 general information questions, asked them to give the answers, asked them whether they were in a TOT state, and then asked them whether they wanted to see the answer. They overwhelmingly wanted to see the answer when they were in a TOT state. This occurred regardless of what their response was, including when they had been correct, had made a commission error, or had made an omission error. Philosophers have sometimes suggested that metacognitive feeling states may be epiphenomenal—having no function in human cognition. By contrast, our results indicate that the special feeling of mild torment that is the hallmark of the TOT state is a goad to epistemic action. It triggers people’s need to know.

## Background

Most studies of the TOT state focus on either the characteristics of the information pertaining to the not-yet-retrieved items—such as the first letter, the number of syllables, the gender of the word in certain languages, or incorrect words called ‘blockers’ (Brown, 1991, 2012; Gollan & Brown, 2006; Kornell & Metcalfe, 2006a; Miozzo & Caramazza, 1997)—or they focus on the compelling phenomenology of the state (Cleary & Claxton, 2015; Schwartz & Cleary, 2016). With respect to the latter, researchers often include William James’ (1890, p. 251) description “The state of our consciousness is peculiar. There is a gap therein; but no mere gap. It is a gap that is intensely active. A sort of wraith of the name is in it, *beckoning us* in a given direction, making us at moments tingle with the sense of our closeness and then letting us sink back without the longed-for term,” R. Brown and McNeill’s (1966, p. 326) comment that people who are “seized by a TOT state … would appear to be in *mild torment*, something like the brink of a sneeze,” or A. Brown’s (1991) acknowledgment of “personal introspections of *inner turmoil* when grappling for an elusive word.” Normally, when individuals are engaged in nonpathological cool cognition, they are not seized by ‘inner turmoil,’ or by any kind of ‘torment.’ But TOT states are different. They arise into consciousness as a distinct emotional configuration that appears to be universally recognizable (Schwartz, 1999). The question that underlies the research in the present article is why this particular metacognitive feeling state surfaces into consciousness as a distinctive and emotional state: what is its function?

Schwartz and Cleary (2016; and see Metcalfe & Schwartz, 2015; Schwartz & Metcalfe, 2011) have suggested that the TOT state may have an adaptive, evolutionary purpose. They have focused on the finding that people persevere in their efforts at recall when they are in a TOT state: the metacognitive feeling appears to prompt people to continue trying. Furthermore, most TOT states are eventually successfully resolved, although with obvious difficulty. Even so, the difficulty of the retrieval itself is thought to play a role in enhancing later memory (see, e.g., Bjork & Linn, 2006)—a consequence that has beneficial effects for the individual’s later performance. The present study takes this idea a step further in suggesting that the TOT state prompts curiosity, which could entail more than just a retrieval attempt—the feeling state may entail general information seeking behavior.

Weiner (2006), in describing the tenants of his attribution theory, argued that consciously experienced feelings (i.e., emotions) are ‘goads to action.’ Consistent with this view, we propose that the conscious emotional instantiation of the TOT state has a function of inducing the experiencer to the action of trying and persisting in efforts to gain the sought-for information. It induces an eager desire to know: curiosity.

Several precursors in the literature provide convergent support for the idea that the TOT state may be related to curiosity and information seeking. First, going back as far as Berlyne’s (1954) theory of epistemic curiosity, theorists have proposed that people’s need to know is ignited by materials that are neither completely unknown nor completely known, but are, instead, in an intermediate zone (Atkinson, 1972; Kornell & Metcalfe, 2006b; Litman, 2009; Loewenstein, 1994; Metcalfe, 2002). These theories indicate that people are best off, as far as learning goes, if they study items that are at an intermediate state of learning. For example, Atkinson (1972) employed an early computerized learning program which sorted items into those that were reliably remembered, intermediate items that were somewhat learned but not permanently (which he called ‘T’ or transitional items), and those that were unlearned. He found that the most effective learning algorithm involved having people selectively study the ‘T’ items. Metcalfe and Kornell (2003, 2005) called T items those in the Region of Proximal Learning (RPL). In addition, they found that college students selectively chose these particular items for further study. Loewenstein (1994), like Berlyne (1954), also zeroed in on such items in his theory of curiosity, pointing to them as being the items people found particularly interesting and deserving of attention.

The items that one finds on the tip of one’s tongue fit the characterization of ‘T’ items or ‘RPL’ items very well. They are not so easy that they can be readily recalled, but they are not so difficult that they are completely unknown and have no resonance. Indeed, some partial and semantic information appears to be retrievable for these items, making them ideal candidates for optimal learning. It is possible that the feeling associated with being in a TOT state is a marker of these special items and has a function of provoking curiosity and inducing the person to epistemic action.

The action involved in information seeking varies from situation to situation. A person may just keep trying to retrieve rather than giving up, may look up an answer in a dictionary, or may ask a friend or teacher. Often Google is the option of choice. To investigate whether the TOT state is perceived as having some special role in inducing such action, we asked 127 participants (61 male, 66 female; mean age = 40.6, SD = 11.6) on MTurk, the following question:

“You have the strongest urge to Google the answer to a question that has been posed to you when:

- you are sure you know the answer, and can produce it,
- you are sure you don’t know the answer and can’t produce it,
- you have a ‘feeling of knowing,’ that is, you think you could probably recognize the answer if it were shown to you.
- you are in a ‘tip-of-the-tongue’ state, that is, you are unable to think of the answer, now, but feel sure that you know it and that it is on the verge of coming back to you.”

Only 25% of our respondents chose the TOT option ‘d’; 39% chose ‘b’ and 31% chose ‘c’. Very few people (5%) wanted to know the answer when they thought they already knew it. Not knowing was associated with information seeking. But people did not appear to prioritize among ‘not known’ states. They said they had the urge to Google when they did not know at all and when they had a feeling of knowing, as frequently as when they were in a TOT state. The mTurk survey, of course, may or may not reflect what people choose to do when actually in such states.

The only empirical investigation that we were able to find of the relation between people’s curiosity and their TOT states was that by Litman, Hutchins, and Russon (2005). They gave participants 12 general-information questions to answer in a self-paced manner, and had them indicate for each whether they knew the answer, they did not know the answer, or they were in a TOT state. They then asked for intensity or confidence ratings. Personality questionnaires were administered, allowing examination of trait curiosity. For some participants a forced-choice recognition test was given. At the end of the experiment, the participants were provided with sealed envelopes with the 12 questions written on the outside and the answers enclosed. The researchers invited participants to open any envelopes they wished. They reported that people were more likely to open the envelopes of questions to which they had been in a TOT state.

Although the results of this experiment are supportive of a link between TOT states and curiosity, by the time the choices were made many, if not most, TOT experiences would probably already have subsided (see Brown & Croft, 2014). To overcome this problem, in the experiment that follows we asked participants whether they wanted to see the answer to the questions the moment after they gave their TOT judgments for each question, while they were still in the state. Furthermore, TOT states are rare—occurring in the natural flow of life (see Brown, 2012) only about once per week, and in a laboratory setting about 10–20% of the time for omission errors. With only 12 questions, there probably were not many TOT experiences per person in Litman et al.’s (2005) study. The frequency was not reported. To accumulate enough TOT experiences to allow more detailed analyses, we used 82 questions rather than just 12. We also speeded responding to each question. TOT experiences often exist in the first few seconds of attempted retrieval, but then, upon successful retrieval, they dissipate. Instead of waiting for this to happen, we pushed people to give a TOT judgment quickly, while they were still in the unresolved state. This meant that the experimenter had to run each participant in person, and enforce timing of the responses. He or she read each question aloud and then allowed only 5 s before the participant had to say whether or not he or she was in a TOT state. Our procedure resulted in a considerably higher proportion of TOT states than is typically observed in self-paced experiments. In addition, we used a load manipulation on half of the questions because working memory load, in some but not all experiments (e.g., Schwartz’s, 2008, Experiments 1, 2 and 3 showed the effect whereas Experiment 4 did not), has been shown to influence the probability of TOT experiences. We wanted to see whether curiosity was evoked by TOT states regardless of whether their overall probability was low or high. The materials were also divided into two sets, because some questions are more likely to evince TOT states than others.

Finally, we asked participants to report TOT states for all responses, even those that were correct. Although most studies look only at TOT experiences when the person has made an omission error, TOT states sometimes occur even when people have made a commission error (they may be in what is sometimes called a ‘blocked’ state, and retrieve the blocker as an error) or even when they were correct (presumably if they had low confidence about their answer). We were interested in all of these TOT states. Our primary question, throughout, was the relation of TOT states to curiosity: did people want to see the answers more when they were in a TOT state than when they were not?

## Experiment

### Method

#### Participants

The 46 participants (20 male, 25 female, one who did not answer) were Columbia University and Barnard College undergraduates, ranging in age from 18 to 29 years (M = 19.96; SD = 2.32). They received partial course credit for participating in the experiment. All procedures were in accordance with the ethical principles of the APA, and were approved by the Columbia University Internal Review Board (IRB-AAAD4902).

#### Materials

The stimuli were 82 general information questions taken from the Nelson and Narens (1980) norms, and updated and corrected (see, Metcalfe, Casal-Roscum, Radin, & Friedman, 2015). For example, one question was “What is the name of the ancient warrior who was dipped in the River Styx?” The questions were displayed on an iMac desktop computer with SuperLab 4.5 software.

#### Design

The experiment was 2 x 2 mixed design, with factors Memory Load (Load or No Load, within participant) and Group (1 or 2, between participants). The questions (Set A) that were in the No Load condition in Group 1 were in the Load condition in Group 2; the questions that were in the No Load condition in Group 2 (Set B) were in the Load condition in Group 1. Twelve questions were reserved to be in the same load conditions in both groups, allowing us to look at overall group differences. On these 12 questions, no significant between-group differences in the probability correct, the probability of commission errors, or the probability of TOT experiences were found. The order of presentation of questions was randomized for each participant.

#### Procedure

Participants were instructed that they would be given a series of general information questions to try to answer and, while trying, to say (a) whether they were in a TOT state or not and (b) whether they wanted to see the answer later or not. They also were informed that they would later be able to see the correct answer for up to 10% of the questions. We included (but did not enforce) this restriction because in pilot testing with no restriction many participants had stated that they wanted to see the answers to all of the questions. The 10% ‘rule’ allowed us to investigate differences depending upon whether people were in a TOT state or not. Participants were told that on half of the questions they would see a string of four digits before the question itself was presented, whereas half of the time they would see asterisks. If a string of numbers appeared they would be required to recall those digits after answering the general information question and giving their judgments. When asterisks appeared, they would later have to say it had been asterisks.

Participants nearly always knew what a TOT state was (also see Schwartz & Metcalfe, 2011). If, however, a participant did not, the experimenter explained it as a state in which “you feel sure you know the answer and think you can get it—it is imminent—but you cannot think of it at the moment.” Participants completed a practice trial before the experiment began.

First, the asterisks or numbers appeared, followed by a 5-s delay. Then, the general information question appeared onscreen and was read aloud by the experimenter, who immediately upon finishing hit a key to start the 5-s response period. During this period, the experimenter typed in the participant’s answer, if any. At the end of the 5 s, participants indicated whether they were in a TOT state or not. The computer then immediately asked whether they wanted to see the answer later. The participant then recalled the four digits or that it had been asterisks, and the program then went on to the next trial.

At the end of the experiment, after filling out a demographics sheet, participants were thanked, debriefed, given course credit for participation, and allowed to see the answers to all of the general information questions.

## Results

In this experiment, people wanted to see the answers more when they were in a TOT state (0.44) than when they were not in a TOT state (0.18), *t*(45) = 8.06, *p* < 0.001. This is the main result of interest, and was obtained in all conditions that we were able to examine, as will be illustrated shortly. There were other differences, however, as indicated below. Data are provided in the Additional file 1.

The probability of a correct answer was 0.26 (SD = 0.15), the probability of a commission error was 0.20 (SD = 0.09), and the probability of an omission error was 0.54 (SD = 0.16). The overall probability of being in a TOT state was 0.24 (SD = 0.15) and all participants exhibited TOT states, but not in all treatment combinations (which will account for differences in degrees of freedom shown in some of the following analyses).

### Omission errors

When we examined the probability of TOT experiences given omission errors, which is the standard analysis, there was no effect of either Group or of Memory Load, but there was an interaction between them, *F*(1, 44) = 9.19, *p* = 0.004, ${\eta}_p^2=0.173$, power = 0.84. This interaction appeared to be due to a difference in TOT experiences for Set A and Set B. Set A was in the No Load condition for Group 1 (with a TOT probability of 0.36) and in the Load condition for Group 2 (*p* = 0.35). Set B was in the Load condition for Group 1 (*p* = 0.28) and in the No Load condition for Group 2 (*p* = 0.30). This same Group × Memory Load interaction was shown in the data on the simple probability of wanting to see later, *F*(1,44) = 4.87, *p* = 0.03, ${\eta}_p^2=0.1$, power = 0.58. People tended to want to see the answers more in the conditions in which there were more TOT experiences.

### Choice to see later when in a TOT state or not (omission errors)

**Fig. 1:**
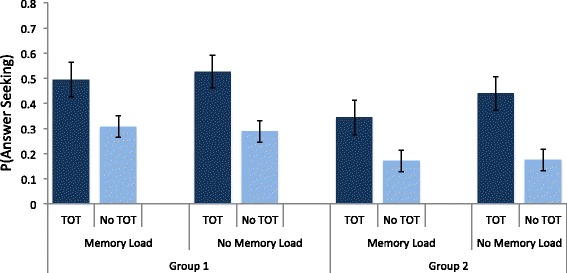
Probability of answer seeking given that the response was an omission error. *Error bars*: standard error of the mean. *TOT*: tip-of-the-tongue

We treated being or not being in a TOT state as if it was an independent variable and computed the conditional probability of choosing to see later as the dependent variable in a 2 (TOT or not) × 2 (Group) × 2 (Memory Load) design. Neither Group nor Memory Load, nor the interaction, was significant. Indeed, the only thing that was significant was whether the person was in a TOT state or not, *F*(1, 43) = 42.68, *p* = 0.001, ${\eta}_p^2=0.50$, power = 1.0. People were about twice as likely to want to see the answer later when they were in a TOT state (0.45) than when they were not (0.24). This difference, as shown in Fig. 1, was evident for every group and for every load condition/question set.

### Commission errors

Commission errors are interesting because TOT experiences sometimes occur when people are in a ‘blocked’ state, or when they have only partial information, such as the first letter. Such responses would have been classified as commission errors here. There were no effects of either Group or of Memory Load, nor was there an interaction between Group and Memory Load.

The simple probability of wanting to see answers later, however, indicated an interaction between Group and Working Memory Load (which may have been a bias toward Set A—as noted earlier), *F*(1, 44) = 3.92, *p* = 0.054, ${\eta}_p^2=0.08$, power = 0.49.

### Choice to see later when in a TOT state or not (commission errors)

**Fig. 2:**
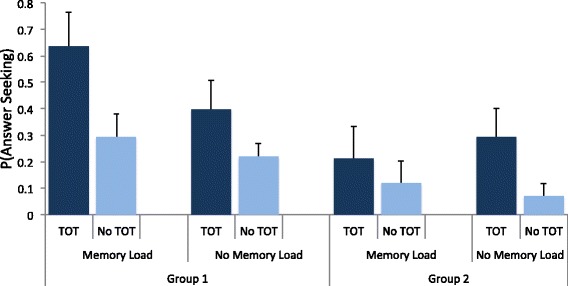
Probability of answer seeking given that the response was a commission error. *Error bars*: standard error of the mean. *TOT*: tip-of-the-tongue

For this analysis, we looked at the subset of data in which the initial response to the question was a commission error—a response that was either partial or wrong (note that ‘don’t know’ responses were counted as omission errors)—and computed the conditional probability of wanting to see later as a function of whether the person was in a TOT state or not, conducting a 2 × 2 × 2 ANOVA with factors TOT, Group, and Memory Load. There was a significant effect of TOT state: people more often chose to see the answer later when they were in a TOT state than when they were not, *F*(1, 19) = 8.44, *p* = 0.009, ${\eta}_p^2=0.308$, power = 0.78. No other effects or interactions were significant. The conditional probability of choice to see later for each of the eight cells for commission errors is shown in Fig. 2.

### Choice to see later when in a TOT state or not (on all errors)

As would be expected, because the separate error-type analyses were significant, when we collapsed over all errors there was a significant main effect for being in a TOT state on choice to see later, *F*(1, 44) = 54.65, *p* < 0.001, ${\eta}_p^2=0.55$, power = 1.0. No other main effects or interactions were significant.

### Correct responses

In this experiment, participants were not given feedback about whether their responses were correct or incorrect, and we observed a number of cases in which people expressed that they were in a TOT state even though they had, in fact, given the correct answer. To our knowledge, this phenomenon has not been observed previously. The dynamics of uncertainty resolution and the feeling of TOT are not well understood. However, it is plausible that a person might feel they were in a TOT state if they were uncertain about their correct response. In that case, these responses, like TOT responses made after either omission or commission errors, should be indicative that people were in their RPL—close to having the right answer—and might evoke curiosity. Accordingly, we examined the correct responses.

There was an effect of Working Memory Load, *F*(1,44) = 8.89, *p* = 0.005, ${\eta}_p^2=0.17$, power = 0.83, showing that people were more likely to be in a TOT state when they had been in the Load condition than in the Asterisks condition. There was also an interaction between Group and Memory Load, *F*(1, 44) = 35.18, *p* < 0.001, ${\eta}_p^2=0.44$, power = 1.0, which was, again, probably due to set difficulty.

### Choice to see later when in a TOT state or not (correct responses)

**Fig. 3:**
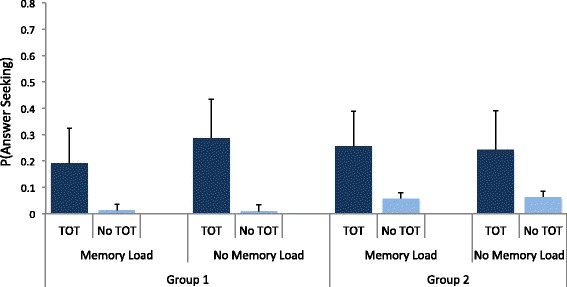
Probability of answer seeking given that the response was correct. *Error bars*: standard error of the mean. *TOT*: tip-of-the-tongue

As found earlier, the only effect that was significant was whether the person was in a TOT state or not, *F*(1, 12) = 8.24, *p* = 0.014, ${\eta}_p^2=0.407$, power = 0.749. People were more likely to want to see the answer later when they had been correct but were in a TOT state (0.24) than when they were correct but not in a TOT state (0.03). This difference, as shown in Fig. 3, was evident for every group and for every load condition/question set for which we had data.

## Conclusion

The results of this experiment indicated that when people were in a TOT state they wanted to see the correct answer more than when they were not in a TOT state. The results of our mTurk survey indicated that people do not realize there is any difference in their own curiosity between merely not knowing and being in a TOT state. Thus, although the TOT state affects people’s pursuit of the answers, people are not aware that it does so. Interestingly, the effect of the TOT state on information seeking occurred at a variety of different levels of TOT experiences, for both groups, and regardless of whether people were in a memory load condition or not. It occurred regardless of the type of error people had made—omission or commission. Indeed, it even occurred when people had been correct. We conclude that the nagging feeling of being in this particular metacognitive state is a goad to epistemic action.

There remain many unaddressed complexities and unanswered questions. Curiosity is often taken to be a pleasant experience—the happy state of an enquiring mind. This perspective, concerning the positive emotional quality of curiosity, contrasts starkly with the nagging, tormented quality so frequently ascribed to TOT states. Interestingly, Litman, Crowson and Kolinski (2010; and see Litman, 2009) have proposed that there may be two kinds of curiosity, which they call interest-based curiosity, which is largely positive, and deprivation-type epistemic curiosity, which is not. The need to know induced by the TOT state examined here appears to be of the latter type, although more studies are needed to investigate the nuances of this emotional distinction. To complicate matters even further, the tormented feeling that accompanies the TOT state prior to resolution may be modulated by anticipation of the highly positive emotionally charged feelings that arise upon solution—feelings of pleasure that may be similar to the delight experienced when a person solves an insight or a magic problem (see, e.g., Danek, Fraps, von Muller, Grothe, & Ollinger, 2014; Hedne, Norman, & Metcalfe, 2016), or experiences a scientific epiphany. The motivation to overcome the torment may go hand in hand with the anticipation of the intense pleasure felt when the state is resolved. Both may contribute to people’s curiosity.

Sometimes metacognitive states have been thought to be epiphenomenal—having no ramifications for cognition. The TOT data presented in this article contravene this conjecture: the presence of the TOT feeling state has implications for people’s choice to seek more information—a propensity with deep consequences for our curiosity, for our engagement in learning, and, ultimately, for our understanding.

## Additional file

Additional file 1: Data. (XLSX 264 kb)

## Abbreviation

TOT: Tip-of-the-tongue
